# A multifunctional ribonuclease A-conjugated carbon dot cluster nanosystem for synchronous cancer imaging and therapy

**DOI:** 10.1186/1556-276X-9-397

**Published:** 2014-08-15

**Authors:** Huiyang Liu, Qin Wang, Guangxia Shen, Chunlei Zhang, Chao Li, Weihang Ji, Chun Wang, Daxiang Cui

**Affiliations:** 1 Institute of Nano Biomedicine and Engineering, Key Laboratory for Thin Film and Microfabrication of Ministry of Education, Department of Instrument Science and Engineering, School of Electronic Information and Engineering, Shanghai Jiao Tong University, Shanghai 200240, People's Republic of China; 2 Research Institute of Translation Medicine, Shanghai Jiao Tong University, Shanghai 200240, People's Republic of China; 3 Department of Biomedical Engineering, University of Minnesota (Twin Cities), Minneapolis, MN 55455, USA

**Keywords:** Carbon dots, RNase A, MGC-803 cell line, Microwave irradiation, Molecular imaging, MTT

## Abstract

Carbon dots exhibit great potential in applications such as molecular imaging and in vivo molecular tracking. However, how to enhance fluorescence intensity of carbon dots has become a great challenge. Herein, we report for the first time a new strategy to synthesize fluorescent carbon dots (C-dots) with high quantum yields by using ribonuclease A (RNase A) as a biomolecular templating agent under microwave irradiation. The synthesized RNase A-conjugated carbon dots (RNase A@C-dots) exhibited quantum yields of 24.20%. The fluorescent color of the RNase A@C-dots can easily be adjusted by varying the microwave reaction time and microwave power. Moreover, the emission wavelength and intensity of RNase A@C-dots displayed a marked excitation wavelength-dependent character. As the excitation wavelength alters from 300 to 500 nm, the photoluminescence (PL) peak exhibits gradually redshifts from 450 to 550 nm, and the intensity reaches its maximum at an excitation wavelength of 380 nm. Its Stokes shift is about 80 nm. Notably, the PL intensity is gradually decreasing as the pH increases, almost linearly dependent, and it reaches the maximum at a pH = 2 condition; the emission peaks also show clearly a redshift, which may be caused by the high activity and perfective dispersion of RNase A in a lower pH solution. In high pH solution, RNase A tends to form RNase A warped carbon dot nanoclusters. Cell imaging confirmed that the RNase A@C-dots could enter into the cytoplasm through cell endocytosis. 3D confocal imaging and transmission electron microscopy observation confirmed partial RNase A@C-dots located inside the nucleus. MTT and real-time cell electronic sensing (RT-CES) analysis showed that the RNase A@C-dots could effectively inhibit the growth of MGC-803 cells. Intra-tumor injection test of RNase A@C-dots showed that RNase A@C-dots could be used for imaging in vivo gastric cancer cells. In conclusion, the as-prepared RNase A@C-dots are suitable for simultaneous therapy and in vivo fluorescence imaging of nude mice loaded with gastric cancer or other tumors.

## Background

Carbon dots (C-dots) are a new member of the carbon nanomaterial family after C_60_, carbon nanotubes, and graphene and were firstly discovered by accident when researchers were trying to purify single-walled carbon nanotubes (SWCNTs) fabricated by arc discharge methods [1]. Since then, many studies concerning C-dots have been reported [2-4]. C-dots have attracted much attention due to their well-defined, nearly isotropic shapes together with their ultrafine dimensions and tunable surface functionalities. Moreover, a variety of simple, fast, and cheap synthetic routes for C-dots have been developed in the past few years including arc discharge, laser ablation, electrochemical oxidation, hydrothermal, combustion/thermal, supported synthetic, and microwave methods [4-6]. Most notable superiority, however, is their potential as replacements for toxic heavy metal-based quantum dots (QDs) which are currently intensively used and are plagued by safety concerns and known environmental hazards [2,5,6].

C-dots have proven themselves in various applications with photoluminescence properties comparable and even superior to those of QDs [2,3,7], such as high photostability, tunable emission, large two-photon excitation cross section [8,9], and non-blinking fluorescence [10]. C-dots have been successfully applied in bioimaging [11], both in vitro [8] and in vivo [12], and even showed significant utility in multiphoton imaging [9]. Moreover, beyond these apparently straightforward applications, more complicated designs aimed at multifunctional nanosystems based on C-dots have been reported. For example, Huang and his collaborators [13] have developed a new theranostic platform based on photosensitizer-conjugated carbon dots with excellent imaging and tumor-homing ability for NIR fluorescence imaging-guided photodynamic therapy. Moreover, a C-dot-based inorganic-organic nanosystem for two-photon imaging and biosensing of pH variation in living cells and tissues has also been designed by Kong's research group [14]. Almost during the same period, C-dots with PEI (polyetherimid)-passivation were used for bioimaging and as nanocarrier for gene delivery [15]. However, with the rapid progress of research and application, many defects were thoroughly exposed such as low photoluminescence intensity, short wavelength excitation, and difficulties in separation and purification, which did hinder it to further in vitro or in vivo biological applications.

Previously, preparation of surfaced-functionalized C-dots usually included three steps: synthesis of raw C-dots, passivation operations, and functionalization reactions [16]. Most C-dots prepared, if without further complicated purification, passivation, and functionality, featured quite low quantum yield (around or less than 5%) [1,17-22] and retained very limited application potentials. So it is extremely necessary to find a simply strategy to fabricate surface-functionalized C-dots with relatively high quantum efficiency.

As to the preparation methods, they could be divided into two categories: top-down methods and bottom-up methods. The bottom-up methods usually suffer from complex processes, or expensive starting materials and severe synthetic conditions, which are unlikely to be extended significantly in the near future [23]. Alternatively, bottom-up synthetic approaches based on chemistry have been desired to achieve C-dots with fluorescence. Presently, Li et al. reported a facile hydrothermal method to prepare luminescent carbon dots (L-CDs) with high quantum yield value (44.7%) and controllable emission wavelengths and used prepared carbon dots to detect toxic Be^2+^ ions [6].

To date, microwave pyrolysis approach, as one family member of bottom-up synthesis methods, has been developed and widely used for its simplicity, cost/time-efficiency, environmental friendliness, easiness to scale up, and more importantly convenience to realize synthesis, passivation, and functionalization reactions simultaneously through only one synthesis step [4,24].

Herein, we report for the first time a green synthesis route, only one synthesis step followed by limited and simple purification, without further passivation and surface functionality to prepare ribonuclease A-conjugated C-dot nanoclusters (RNase A@C-dots). It is well known that RNase A is a low molecular weight protein (approximately 124 residues, approximately 13.7 kDa, pI = 9.4) with a globular conformation (2.2 nm × 2.8 nm × 3.2 nm) [25]. The protein has proved to be thermally stable [26], even under microwave heating for a couple of minutes [27]. In addition, RNase A has been confirmed to be able to inhibit the growth of cancer cells as a result of its enzymatic activity of degrading cellular RNA and has been proven to be effective in clinical trials as a chemotherapeutic agent [28,29]. In our previous work, we used RNase A as a biomolecular templating agent to synthesize CdTe QD nanoclusters [27]. Meanwhile, through chemical bonding of the targeting RGD peptide on the RNase A@CdTe QD cluster surface, we constructed multifunctional biological nanoprobes which shows the efficiency of the nanosystem for synchronous in vitro targeted cancer imaging and therapy [27]. Inspired by the achievements of previous studies and concerned with the shortcomings along with the accomplishments, we proposed the synthesis of RNase A@C-dots via a one-step microwave-assisted method using citric acid as carbon precursor and RNase A as an assisting agent. The method greatly simplified the synthesis processes, conveniently realized the improvement of the photoluminescence intensity, and largely retained the activity of RNase A for potential therapeutic applications. Prepared RNase A@C-dots exhibited multifunctional properties and were successfully employed for tumor fluorescence imaging and therapy.

## Methods

### Materials

Bovine pancreatic ribonuclease A (RNase A) and polyethylene glycol (PEG_2000N_) were purchased from Sigma-Aldrich Chemical Co. (St. Louis, MO, USA). Citric acid (CA, analytical grade) was bought from Shanghai Chemical Reagent Co., Ltd. (Shanghai, China). 3-[4,5-Dimethylthiazol-2yl]-2,5-diphenylterazolium bromide (MTT) was obtained from Invitrogen Corporation (Carlsbad, CA, USA). MGC-803 cell lines were obtained from the Cell Bank of Type Culture Collection of Chinese Academy of Sciences. Cell culture products and reagents, unless pointed out, were all purchased from Gibco (Invitrogen Corporation, Carlsbad, CA, USA). All chemical reagents were used without further purification. All solutions were made with purified water (with a low electroconductivity of 18.2 MΩ cm).

### Synthesis of RNase A@C-dots, C-dot, and C-dots-NH_2_ (C-dot surface modified by PEG_2000N_)

For the synthesis of RNase A@C-dots, 2 g citric acid and 0.15 g RNase A were diluted in 10 ml water within a 25-ml glass bottle and put under ultrasonic for 1 to 2 min to form a uniform solution. Then, the transparent solution was put into a domestic microwave oven (700 W) for 3 to 5 min. After cooling to room temperature, the obtained brown C-dot solution was dialyzed against pure water with a dialysis membrane (molecular weight cutoff (MWCO) of 1,000) for 2 days to remove unreacted citric acid. Finally, the dry C-dot composite was freeze-dried in vacuum, weighed, and dissolved in ultrapure water with a fixed concentration.

In control experiments, citric acid without RNase A was treated with the same procedure and the final product was named C-dots. Then, to modify the surface of C-dots with PEG_2000N_, the mixture solution of C-dots and PEG_2000N_ (mass ratio, C-dots/PEG_2000N_ =1:20) was refluxed with nitrogen gas protection at 120°C for 72 h. Then, the mixture was shifted into a dialysis membrane (MWCO of 3,000) against pure water to remove surplus PEG_2000N_.

### Characterization

To determine the size and morphology, RNase A@C-dots were characterized by high-resolution transmission electron microscopy (HR-TEM, JEM-2100 F, 200 kV, JEOL Ltd., Tokyo, Japan). The samples for TEM/HR-TEM were made by simply dropping aqueous solution of the C-dots onto a 300-mesh copper grid casted with a carbon film. UV–Vis absorption spectra of the C-dots were measured with a Varian Cary 50 spectrophotometer (Varian Inc., Palo Alto, CA, USA). Fluorescence excitation and emission spectra of RNase A@C-dots were recorded on a Hitachi FL-4600 spectrofluorimeter (Hitachi Ltd., Tokyo, Japan). Zeta potential of RNase A@C-dots was measured on a Nicomp 380 ZLS zeta potential/particle sizer (PSS. Nicomp, Santa Barbara, CA, USA). X-ray photoelectron spectroscopy (XPS) was obtained at room temperature by a Kratos Axis Ultra spectrometer (AXIS-Ultra DLD, Kratos Analytical Ltd., Tokyo, Japan) using a monochromated Al Kα (1486.6 eV) source at 15 kV. Fourier transform infrared (FTIR) spectra were obtained on a Nicolet 6700 spectrometer (Thermo Electron Corporation, Madison, WI, USA). The samples for FTIR measurement were prepared by grinding the dried C-dots with KBr together and then compressed into thin pellets. X-ray diffraction (XRD) profiles of the C-dot powders were recorded on a D/MAX 2600 PC (Rigaku, Tokyo, Japan) equipped with graphite monochromatized Cu Kα (*λ* = 0.15405 nm) radiation at a scanning speed of 4°/min in the range from 10° to 60°. Time-resolved fluorescence intensity decay of RNase A@C-dots was performed on a LifeSpec II (Lifetime only, Edinburgh Instruments, Livingston, UK). The sample was excited by 380-nm laser, and the decay was measured in a time scale of 0.024410 ns/channel.

### Quantum yield measurement

To assess the quantum yield of RNase A@C-dots, quinine sulfate in 0.1 M H_2_SO_4_ (quantum yield, 54%) was used as a reference fluorescence reagent. The final results were calculated according to Equation 1 below:

(1) $$\Phi_{\mathrm{sample}}=\frac{A_{\mathrm{std}}}{A_{\mathrm{sample}}}\times \frac{F_{\mathrm{sample}}}{F_{\mathrm{std}}}\times \frac{n_{\mathrm{sample}}^{2}}{n_{\mathrm{std}}^{2}}\times \Phi_{\mathrm{std}}$$

where Φ_std_ is the known quantum yield of the standard compound, *F*_sample_ and *F*_std_ stand for the integrated fluorescence intensity of the sample and the standard compound in the emission region from 380 to 700 nm, *A*_std_ and *A*_sample_ are the absorbance of the standard compound and the sample at the excitation wavelength (360 nm), and *n* is the refractive index of solvent (for water, the refractive index is 1.33).

To minimize the reabsorption effects, UV absorbance intensities of the samples and standard compound should never exceed 0.1 at the excitation wavelength. Photoluminescence (PL) emission spectra of all the sample solutions were measured at the excitation wavelength of 360 nm. The integrated fluorescence intensity is the area under the PL curve in the wavelength from 380 to 700 nm.

### Cell culture, cell viability assay, and time-dependent cell response profiling

The human gastric cancer cell line MGC-803 cell was maintained at 37°C (5% CO_2_) in Dulbecco's modified Eagle's medium (DMEM, HyClone, Thermo Fisher Scientific, Waltham, MA, USA) supplemented with 10% (*v*/*v*) fetal bovine serum (Gibco), 100 U/ml penicillin, and 1 mg/ml streptomycin.

For MTT assay, MGC-803 cells were seeded in a 96-well plate (Corning Costar, Corning, NY, USA) with a density of 5 × 10^3^ cells/well with 10% fetal bovine serum and then cultured overnight. After culturing, those cells were incubated with C-dots of various concentrations for 24 h. Following the incubation, the supernatant was removed and the cells were washed once with 0.01 M PBS. Then 150 μl DMEM and 15 μl MTT stock solution (5 mg/ml in PBS, pH 7.4) were added to each well, and after this, the cells were allowed to incubate for 4 h at 37°C. Finally, after removing the culture medium, 150 μl DMSO was added to dissolve the Formosan crystals. The optical density (OD) was measured at 570 nm on a standard microplate reader (Scientific Multiskan MK3, Thermo Fisher Scientific, Waltham, MA, USA). The cell viability was calculated according to the following formula: Cell viability = (OD of the experimental sample/OD of the control group) × 100%. The cell viability of control groups was denoted as 100%.

The time-dependent cell response profiles were performed using a real-time cell electronic sensing (RT-CES) system. Firstly, 100 μl of media was added to 16-well E-plates to record background readings, and then, 100 μl of cell suspension (containing about 5,000 cells) was added. Secondly, the cells in the E-plates were allowed to incubate at room temperature for 30 min. After the incubation, the E-plates were put on the reader in the incubator to continuously record the electric impedance which is reflected by cell index. After 20 to 24 h, the RNase A@C-dots and C-dots of certain concentration were added into the E-plates to mix with cells. For comparison, each plate also contained wells added with RNase A and wells with cells alone in the media in addition to media-only wells. The cells were monitored every 2 min for the first 1 h after the addition of C-dots and RNase A to get the short-term response and for every 30 min from 1 h after C-dot addition to about 48 h to record the long-term response.

### Laser scanning confocal microscopy imaging in vitro

For fluorescence imaging with RNase A@C-dots, MGC-803 cells were first plated on 14-mm glass coverslips and allowed to adhere for 24 h at 37°C. Second, the cells were co-incubated with 120 μM RNase A@C-dots for 24 h. Then, the cells were washed with phosphate buffered (PBS) solution to remove unbound nanoparticles. Finally, the cells were fixed with 4% paraformaldehyde, and the nuclei of the cells were stained with 4′,6-diamidino-2-phenylindole (DAPI) (0.5 mg/ml in PBS).

Confocal fluorescence 2D or 3D imaging was performed on a TCS SP5 confocal laser scanning microscope (Leica Microsystems, Mannheim, Germany) with an objective lens (×63). In our experiment, we used a 408-nm excitation wavelength laser. Optical sections were averaged three times to reduce noise.

### RNase A@C-dots for in vivo fluorescence imaging

Male 4-week-old athymic nude mice were purchased from Shanghai Slac Laboratory Animal Co. Ltd (Shanghai, China). All experiments that involve animal use were performed in compliance with the relevant laws and institutional guidelines. All animal experiments were approved by the Institutional Animal Care and Use Committee of Shanghai Jiao Tong University (No. SYXK2007-0025). For the establishment of the tumor model, MGC-803 cells were resuspended in PBS, and 2 × 10^6^ cells per site were subcutaneously injected. The tumor nodules had reached a volume of 0.1 to 0.3 cm^3^ approximately 3 weeks post-injection. For in vivo fluorescence tumor imaging experiments, 100 μl (5 mg/ml) RNase A@C-dot aqueous solution was intratumorally injected into the MGC-803 tumor-bearing mice. Time-course fluorescent images (excitation, 500/20 nm; emission, 600/30 nm; integration time, 5 s) were acquired on a Bruker In-Vivo F PRO imaging system (Bruker, Billerica, MA, USA).

## Results and discussion

### Characterization and properties of RNase A@C-dots

**Figure 1:**
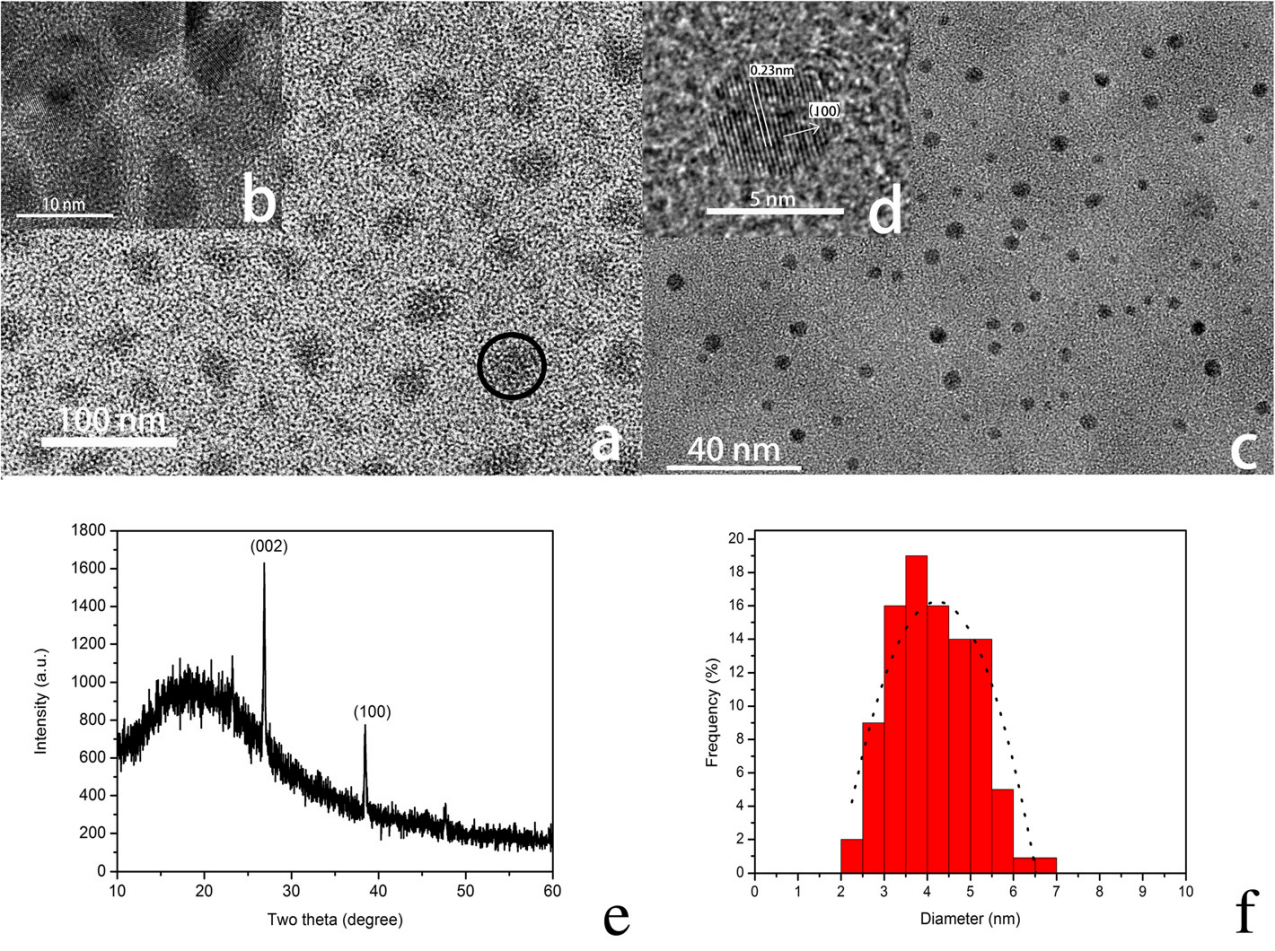
**TEM and HR-TEM images, XRD pattern, and size distribution of RNase A@C-dots. (a)** TEM image of the as-prepared RNase A@C-dots inside the dialysis membrane after dialyzing against pure water. One typical RNase A@C-dot cluster is labeled with a black circle. **(b)** High-resolution TEM (HR-TEM) image of one focused area within the black circle. **(c)** TEM image of the C-dots outside the dialysis membrane. **(d)** HR-TEM image of one single C-dot. **(e)** XRD pattern of RNase A@C-dots. **(f)** Size distribution of C-dots.

TEM images of the as-prepared RNase A@C-dots that were trapped in the dialysis membrane (MW cutoff 1,000) are shown in Figure 1a; the size of the RNase A@C-dots varies mainly within 25 to 45 nm with relatively irregular morphologies. High-resolution TEM image (Figure 1b, the zoomed-in image of the area within the circle in Figure 1a) clearly shows that the particles are actually formed by encapsulating several C-dots within the RNase A film, so we can call them clusters. The clusters can also extremely easily disperse in pure water. In Figure 1c, the average size of C-dot that dispersed out of the dialysis membrane is about 4 nm (Figure 1f) in diameter with nice spherical morphologies (Figure 1d), and the dispersions are also excellent. Lattice spacing of approximately 0.23 nm clearly displayed in the high-resolution TEM image (Figure 1d) indicates the (100) facet of graphite [30].

We can reasonably conclude that during the reaction process accelerated by microwave heating, RNase A capped the different numbers of C-dots that cause the different sizes of particles. When dialyzing against pure water, the smaller single particles diffused out of the dialysis membrane (MW cutoff 1,000), while the larger sized clusters remained inside the membrane. So we can collect two kinds of the relative uniform particles. In our experiments, we use the RNase A@C-dot clusters that are retained in the dialysis membrane except for special description.

As shown in Figure 1e, the XRD pattern of the RNase A@C-dot clusters has two distinctly sharp peaks at 2*θ* of approximately 27° (*d* = 0.33 nm) and approximately 39° (*d* = 0.23 nm) which can be attributed to (002) and (100) facets of graphite [30]. Notably, there is a broad peak at 2*θ* of around 20° (*d* = 0.42 nm) which is probably the reflection of the (002) facet of graphite; however, the larger interlayer spacing of 0.42 nm compared to that of bulk graphite which is about 0.33 nm might have resulted from the poor crystallization [31].

**Figure 2:**
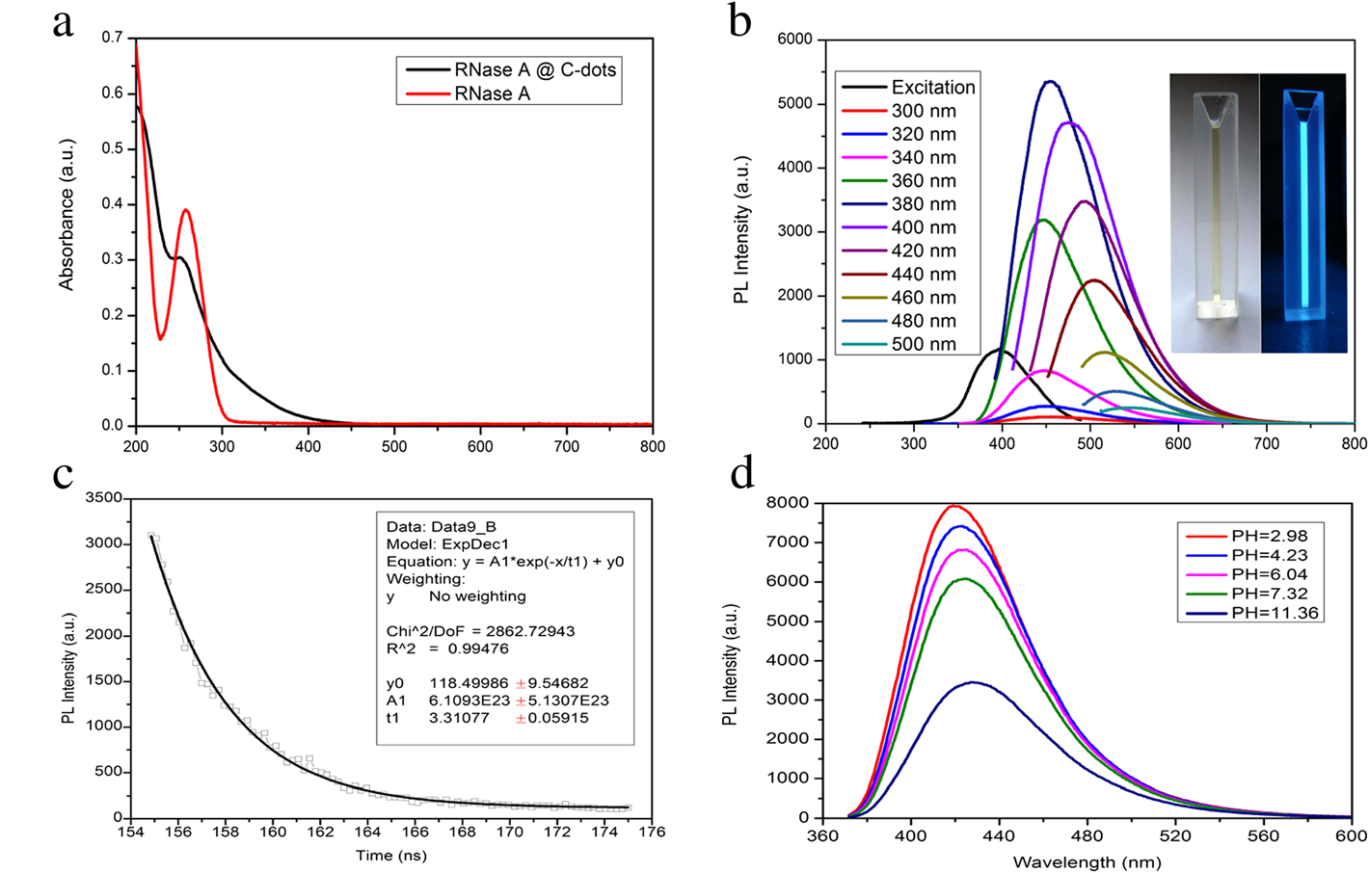
**UV–Vis absorption and PL spectra and fluorescence decay profile of RNase A@C-dots. (a)** UV–Vis absorption of the as-prepared RNase A@C-dots (black line) and RNase A treated by microwave for 4 min (red line). **(b)** PL spectra of the as-prepared RNase A@C-dots at excitation from 300 to 500 nm in 20-nm increment. Inset: image of the as-prepared RNase A@C-dot dispersion under visible light (left) and UV light (right). **(c)** Fluorescence decay profile (*λ*_ex_ = 380 nm, *λ*_em_ = 450 nm) of the as-prepared RNase A@C-dots. **(d)** The effect of the solution pH value over the fluorescence (*λ*_ex_ = 360 nm) of the as-prepared RNase A@C-dots.

The UV–Vis absorption spectra (Figure 2a, black line) of the RNase A@C-dots feature a typical absorbance of C-dots which shows strong optical absorption in the UV region with a tail extending out into the visible range [8]. On the other hand, the absorbance peak of the pure RNase A is at approximately 275 nm as shown in Figure 2a (red line). Compared with UV–Vis absorbance peaks of C-dots (prepared by microwave synthesis using citric acid as a carbon precursor without RNase A) and the pure RNase A, there are clearly differences in UV–Vis absorption spectra. First, the absorbance peak of the C-dots (Additional file 1: Figure S2a) is at approximately 240 nm which has resulted from π-π* transition [32], while in the absorbance spectrum of RNase A@C-dots (Figure 2a, black line), the peak shifts to approximately 260 nm which may be caused by the increasing size of RNase A@C-dots as a cluster and the synergy of RNase A and C-dots. In the TEM image of C-dots, it has shown clearly that the RNase A@C-dots are actually clusters with several C-dots capped by RNase A films. The RNase A itself did not distinctly change its UV–Vis absorption character before and after microwave treatment for 4 min (see Additional file 1: Figure S1). Second, there is a noticeable absorbance increase of RNase A@C-dots from 300 to 450 nm compared to that of C-dots which is very likely to benefit from the surface passivation by RNase A [24].

Dramatic changes have been reflected in their PL properties. The PL spectrum of RNase A@C-dots (Figure 2b) shows a clear *λ*_ex_ dependence of both emission wavelength and intensity just as we have reported before [8]. Specifically, as the excitation wavelength changes from 300 to 500 nm in a 20-nm increment, the PL peak shifted from 450 to 550 nm, while the intensity increases before the excitation wavelength reaches 380 nm and then gradually decreases followed by increase of excitation wavelength. However, in the PL spectra of C-dots (Additional file 1: Figure S2b), we cannot find that there is no a typical *λ*_ex_ dependence character. When the excitation wavelength changes from 280 to 440 nm, the PL intensity at around 480 nm varies and hits its maximum at an excitation wavelength of 380 nm. But the emission wavelength does not change its location. Moreover, before the excitation wavelength reaches 380 nm, there is more than one emission peak in the PL spectra with only one peak around 480 nm remaining when excited at 390 nm and longer wavelength. Furthermore, photoluminescence excitation (PLE) spectra of RNase A@C-dots (Figure 2b) have only one peak located at around 390 nm, while the PLE spectra of C-dots (Additional file 1: Figure S2b) owns two with an additional one around 290 nm.

The existence of RNase A has not only changed the features and locations of PL spectra but also enhanced the intensity of photoluminescence. When excited at 360 nm, the intensity of RNase A@C-dots is about 30 times the intensity of C-dots (Additional file 1: Figure S2c). As to quantum yield, Table 1 shows that the quantum yield of the RNase A@C-dots is 24.20% which is dramatically higher than the 0.87% yield of C-dots. Even after having been passivated with PEG_2000_ which is widely accepted as an efficient way to improve the quantum yield of C-dots [8], the quantum yield of C-dots is 4.33%, still much lower than that of the RNase A@C-dots.

**Table 1:** **Related photoluminescent quantum yield (PLQY) of RNase A@C-dots, C-dots, and C-dots-PEG**_**2000 **_**(C-dots passivated by PEG**_**2000**_**)**

**Sample**	**RNase A@C-dots**	**C-dots**	**C-dots-PEG**_**2000**_
PLQY [%]	24.20	0.87	4.33

Luminescence decay (Figure 2c) has an average excited-state lifetime of 3.3 ns for emission at 450 nm with an excitation wavelength of 380 nm which is comparable to those reported [2,23]. The relatively short lifetime might as well suggest the radioactive recombination of the excitation contributing to the fluorescence [23].

**Figure 3:**
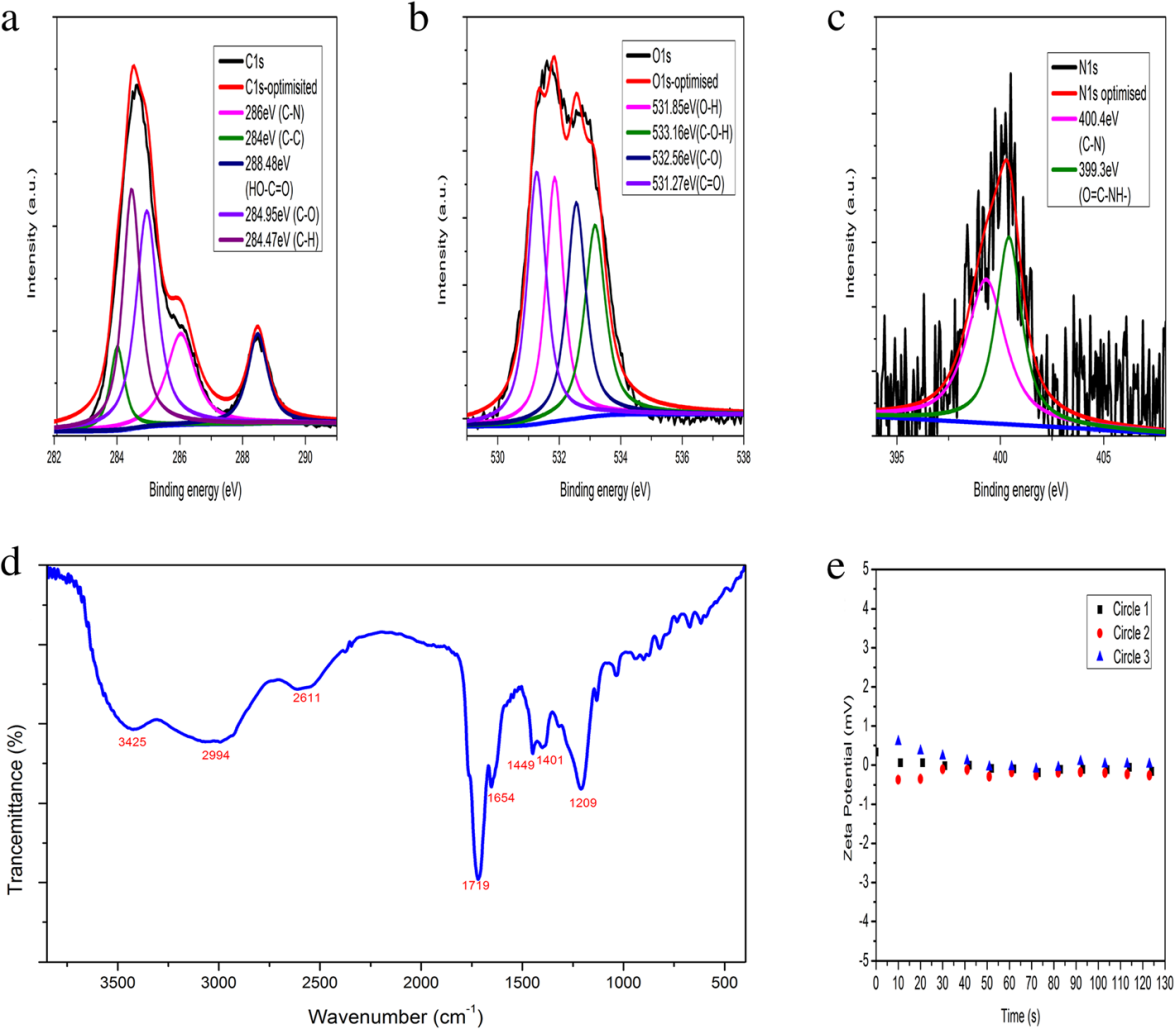
**XPS and FTIR spectra and zeta potential. (a)** XPS C 1 s spectrum. **(b)** XPS O 1 s spectrum. **(c)** XPS N 1 s of RNase A@C-dots. **(d)** FTIR spectra of RNase A@C-dots. **(e)** Zeta potential of RNase A@C-dots.

The FTIR spectrum (Figure 3d) shows the presence of (C = O) (1,719 cm^−1^), (O-H) (3,425 cm^−1^), (C-N) (1,209 cm^−1^), and (N-H) (2,994 cm^−1^) which directly indicates Rnase A coated C-dot surface. This can also be confirmed by the X-ray photoelectron spectroscopy (XPS) of RNase A@C-dots (as shown in Figure 3a,b,c). Moreover, the high-resolution N 1 s spectrum of the RNase A@C-dots (Figure 3c) has clear signs of both amide N (399.3 eV, C-N) and doping N (400.4 eV, O = C-NH-) atoms. The XPS (Additional file 1: Figure S3) of the C-dots only shows the signals of -COOH and -OH, and neither amide N nor doping N is detected. So we may speculate that the RNase A might have played double roles in the microwave-assisted synthesis process: N doping and surface passivation. They are responsible for the enhanced PL intensity of RNase A@C-dots [33].

The average zeta potential of C-dots (Figure 3e) is 0.02 mV, slightly beyond zero. Considering the fact that cells are with positive charges, a zeta potential of no less than zero is definitely favorable in cell labeling and imaging. (The influence of microwave condition on PL of carbon dots was also investigated, as shown in Additional file 1: Figure S5).

### Effects of pH on PL properties of RNase A@C-dots

Although the mechanism of PL properties of C-dots is still unclear and debatable, there is solid evidence of lower quantum efficiency of C-dots that is caused by the fast recombination of excitations located at surface energy traps [8]. Therefore, after modifying the surface of C-dots using different surface passivation reagents, the PL properties of the C-dots can be significantly improved [7,8,34]. In this work, we firstly introduce the bioactive enzyme RNase A to synthesize C-dots by one-step micro-assisted synthesis method. The mechanism of the PL enhancement could be explained by following two reasons: Firstly, we propose that the electron-donating effect which resulted from the abundant amino acid groups on the surface of RNase A, especially those amino acids with benzene rings, might contribute a lot to the much enhanced PL intensity of the C-dots. To test our assumption, we select tryptophan and thenylalanine as replacements of RNase A to synthesize C-dots in the same conditions. As shown in Additional file 1: Figure S5b, both tryptophan and thenylalanine can greatly enhance the PL intensity. Secondly, we think that in the microware heating reaction, RNase A acts as a N doping reagent that causes the PL enhancement of the C-dots. The data of IR and XPS can also support the point.

In the biological application, pH is a very important factor that we firstly take into consideration. Herein, the influence of pH values over the PL of the RNase A@C-dot clusters is indicated in Figure 2d. The fact that pH values could affect the PL intensity has been seen in quite a few studies [10,21,32,35]. Generally, PL intensity reaches its maximum at a certain pH values, 4.5 [35] or 7 [21]. At the same time, a slight redshift in the emission peak was identified with the increase of pH value [35]. Interestingly, the pH value played a unique role upon the PL of RNase A@C-dots. There was a noticeable redshift in the emission peak when the pH went from 2.98 to 11.36. However, the PL intensity decreases continuously as pH values increase. Specifically, the C-dots lost about 25% of its PL intensity when the pH increases from 2.98 to 7.32 and retain only 40% of its intensity when the pH value comes to 11.36. We speculate that at the lower pH condition, the RNase A coating on the surface of C-dots keeps a high activity and the RNase A-modified C-dots remain monodispersed in the water solution. Although the underlying origin is still vague, the fact that the C-dots keep its PL intensity at a relatively high level, going through the pH value from very acidic to neutral, shows promising advantages in biological applications.

### Laser scanning confocal microscopy imaging in vitro

**Figure 4:**
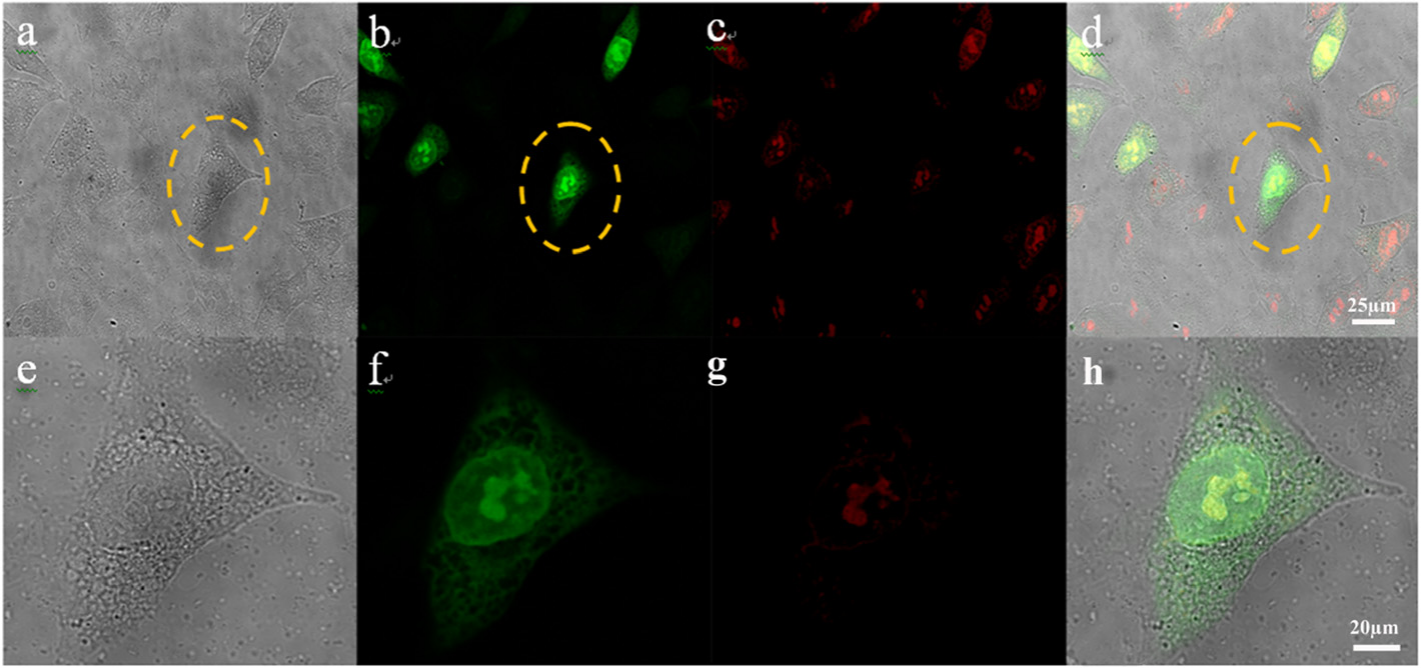
**Laser scanning confocal microscopy images of MGC-803 cells. (a)** Picture of MGC-803 cells under white light. **(b)** Picture of MGC-803 cells under excitation at 405 nm. **(c)** Picture of MGC-803 cells under excitation at 536 nm. **(d)** Overlapping picture of MGC-803 cells under excitation at 405 and 536 nm. **(e)** Amplified picture of a single MGC-803 cell under white light. **(f)** Amplified picture of a single MGC-803 cell under excitation at 405 nm. **(g)** Amplified picture of a single MGC-803 cell under excitation at 536 nm. **(h)** Overlapping picture of a single MGC-803 cell under excitation at 405 and 536 nm.

**Figure 5:**
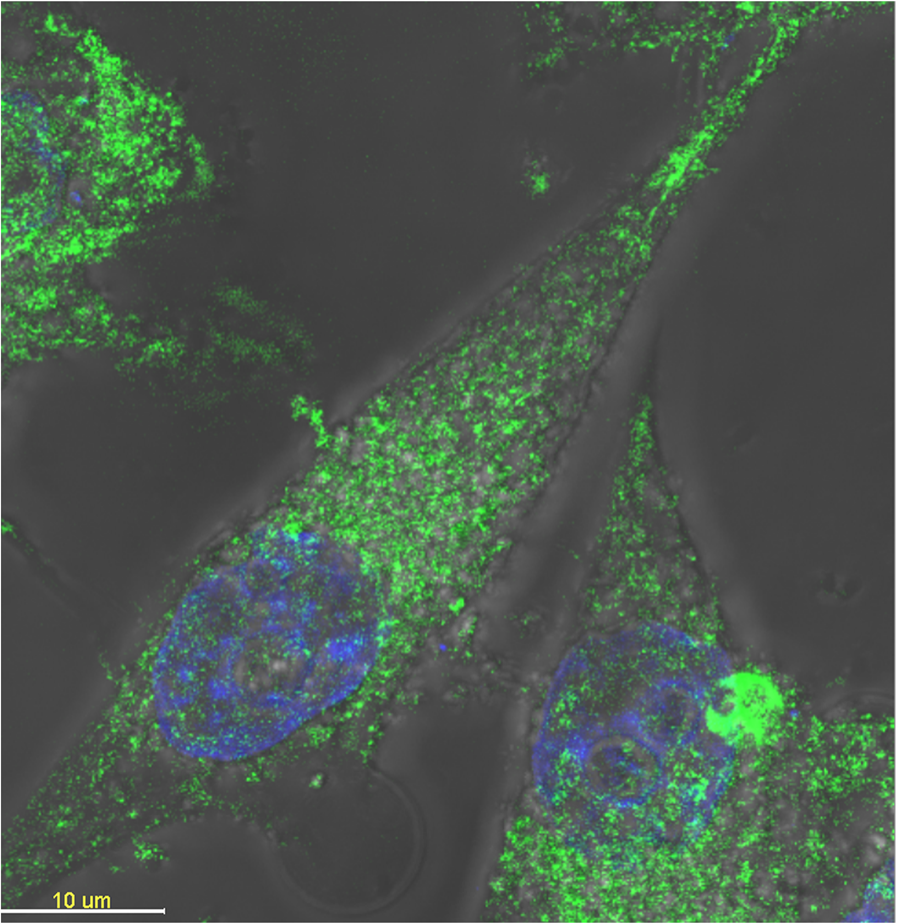
Laser scanning confocal microscopy images (3D mode) of MGC-803 cells.

Figure 4 shows the 2D images of MGC-803 cells labeled with RNase A@C-dots. After co-incubation with RNase A@C-dots, MGC-803 cells show bright green color over the entire cell upon excitation at 405 nm. The nuclei marked by PI, when excited at 536 nm, featured strong red fluorescence. A merge image clearly shows that the RNase A@C-dots can enter the cell via the endocytic route. Moreover, we can also find that in up to 10% cells, there are clearly green dots existing in the nucleus. Meanwhile, a 3D confocal imaging (Figure 5) of the cell clearly reveals that the RNase A@C-dots have entered the cell, while the carbon dots reported before [7] were mostly in the cytoplasm and membrane, with only minor penetration into the cell nucleus. Until now, we can give an explanation for the transportation into the nucleus. It may be caused by the small size of RNase A@C-dots which enables perfect dispersion or assists protein (derived from RNase A) action.

### Cytotoxicity assay by MTT and real-time cell electronic sensing

**Figure 6:**
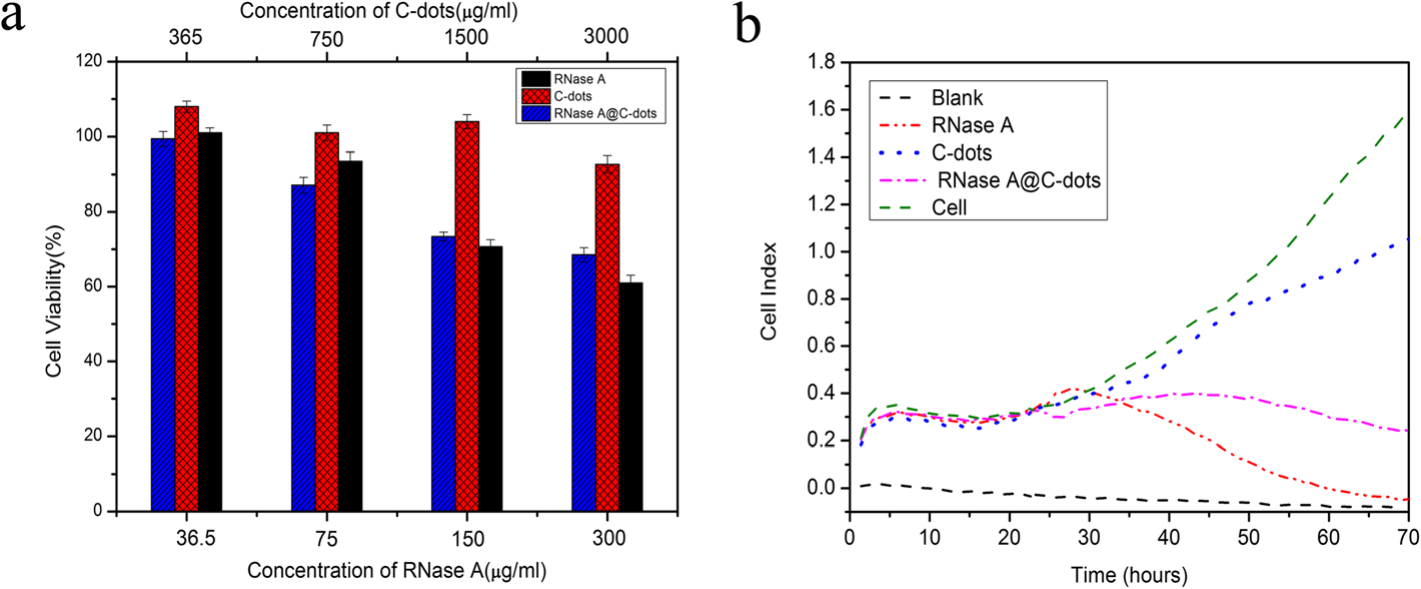
**Cytotoxicity assay results. (a)** MTT assay determined cytotoxicity profile of RNase A, C-dots, and RNase A@C-dots after 24 h incubation with MGC-803 cells at varied concentrations (sample size *N* = 3). **(b)** Dynamic monitoring cytotoxic response of MGC803 cells to RNase A, C-dots, and RNase A@C-dots.

To test the potential of the RNase A@C-dots in cancer therapy, MTT assay was used to determine the cytotoxicity profile. The different concentrations of RNase A@C-dots were incubated with MGC-803 cells, respectively, for 24 h at 37°C. In control experiments, we select RNase A and C-dots to carry out accordingly the same procedure and keep equal contents of bare C-dots with RNase A@C-dot solution. The results (Figure 6a) show clearly that RNase A alone could restrain the cancerous cells due to the ribonuclease-mediated toxicity [27]. Moreover, the ability of RNase A in inhibiting the cancerous cells exhibits a content-dependent character with a relatively low cell viability (61%) at higher concentration (300 μg/ml) and a high one at lower concentration (36.5 μg/ml). Noting the fact that the bare C-dots demonstrate no cytotoxicity (cell viability 92.7%) even at the highest concentration (3,000 μg/ml), we could conclude reasonably that the low cell viability (68.5%) which resulted from C-dot treated at the same concentration comes not from the bare C-dots but from RNase A on the surface via its ribonuclease-mediated toxicity. So, by MTT assay, we have validated the potential of the RNase A@C-dots in cancer therapy.

While MTT assay only gave the information of cytotoxicity at fixed time points, the time-dependent cell response profiling was performed using real-time cell electronic sensing (RT-CES). Without cell labeling, the RT-CES assay directly reflected changes in cell biological status including cell viability, cell number, morphology, and adhesion [36]. Briefly, increasing in cell adhesion or cell spread will result in a larger cell/electrode contact area which is presented by a larger cell index (CI) value, while on the other hand, toxicity induced cells can round up leading to a smaller CI [37]. In good accordance with the MTT, RNase A (150 μg/ml) or RNase A@C-dots with the same RNase A concentration (150 μg/ml) were used based on the results of MTT assay. RNase A alone can inhibit and kill cancerous cells with a final CI value very close to zero (Figure 6b), and RNase A@C-dots also show competent ability in killing cancer cells with a CI value of around 0.2 compared to 1.8 of cells alone. In fact, RNase A and C-dots featured some differences concerning their performances. After adding of RNase A, the CI value increased a little bit in the first 4 h and then decreased continuously, while the adding of RNase A@C-dots resulted in a nearly unchanged CI value until about 50 h and then a decrease in CI value until the end. We suggest that this might have resulted from the difference of migration rate. In order to inhibit the cancerous cells, RNase A must enter cells and mount to a certain concentration. Suffering from a lower migration rate, it takes more time for RNase A@C-dots to concentrate into the cells compared to RNase A. As expected, RNase A@C-dots could hardly be considered as toxic as the CI value kept increasing at the beginning of nearly 50 h. However, after 50 h, the CI value became lower than that of the control group. This may be caused by the concentration accumulative effect of RNase A@C-dots in the cells which could have an impact over the status of the cells within an acceptable range. So it is proven that the RNase A@C-dots could kill cancerous cells with its ribonuclease-mediated toxicity from surface-doped RNase A, and not C-dots itself. However, the dose of the RNase A or RNase A@C-dots is a key factor to have an effect on the therapy, and the specific mechanism is still unclear and further study is necessary.

### RNase A@C-dots for in vivo imaging of gastric cancer

**Figure 7:**
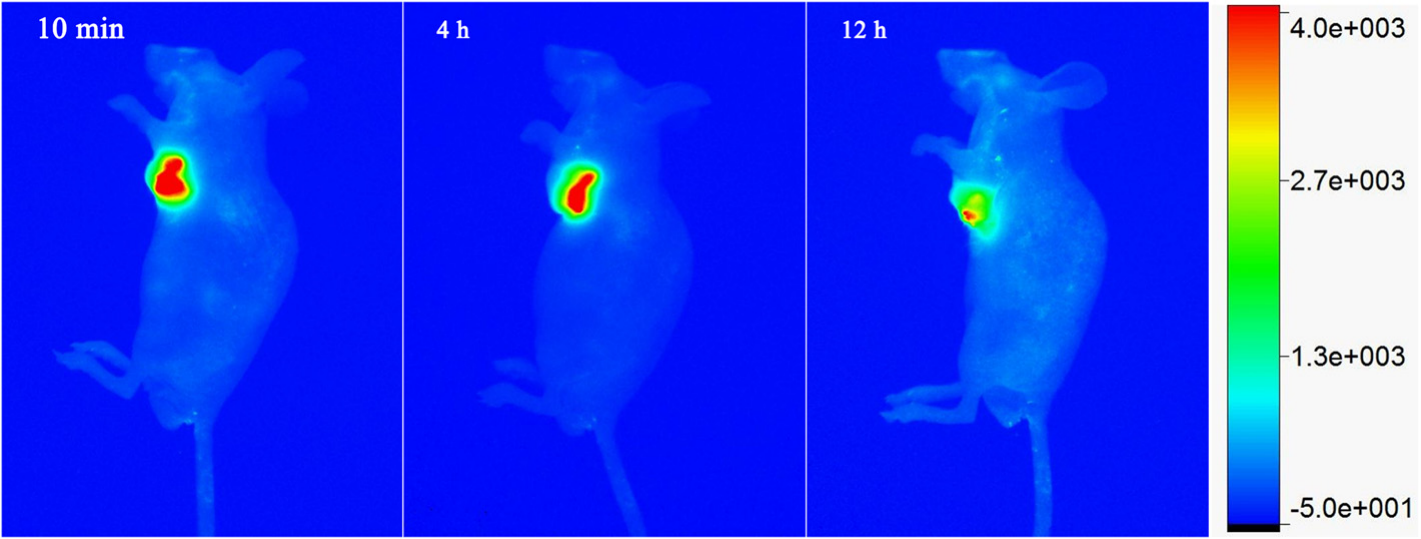
**Representative in vivo fluorescence images of MGC-803 tumor-bearing mouse.** After intratumoral injection with RNase A@C-dots after 10 min, 4 h and 12 h.

As shown in Figure 7, obvious luminescence signal could be observed in the tumor after intratumoral injection. The RNase A@C-dots resulted in high contrast images and could be easily distinguished from the background. The luminescence intensity shows a clear time-dependent characteristic. Twelve hours after injection, the luminescence intensity had been dramatically decreased. This could probably be explained by the ability of carbon dots to pass the glomerulus and be excreted by urine [38].

## Conclusions

In summary, we have synthesized the multifunctional RNase A@C-dots particles by one-step microwave method using the biological molecule of RNase A as an assistant reagent. The RNase A@C-dots show much enhanced fluorescence intensity in contrast to bare C-dots. The quantum yield is nearly 30 times higher reaching 24.20% instead of 0.87% with a narrow Stokes shift only of approximately 80 nm. The RNase A@C-dots could not only penetrate the cell membrane but can also enter the nuclei of cells efficiently. Moreover, the RNase A@C-dots also show potential ability in inhibiting and killing cancer cells. Hopefully, the RNase A@C-dots could be used in nanodiagnostics and nanotherapeutics in the feature. But before that, the detailed mechanism which still remains vague behind the interactions between the C-dots and cancer cells should be fully understood.

### Supporting information

Supporting information is available from the XX Online Library or from the author.

## Competing interests

The authors declare that they have no competing interests.

## Authors’ contributions

LH carried out the preparation and characterization of RNase A@C-dots and drafted the manuscript. WQ finished the MTT test. ZC finished the gastric cancer-bearing animal model preparation. LC and JW finished the RNase A@C-dots intratumor injection and imaging experiment. SG, WC, and CD designed and coordinated all the experiments. All authors read and approved the final manuscript.

## Supplementary Material

Additional file 1 **Supplementary figures.** A document showing six supplementary figures showing UV–Vis absorption of RNase A, PL and XPS spectra of C-dots, and influence of ratio reactants, reaction time, carbon sources, and surface modification molecules on the PL character of RNase A@C-dots. Click here for file
