# Supplementary material for: A multifunctional ribonuclease A-conjugated carbon dot cluster nanosystem for synchronous cancer imaging and therapy
Source: Nanoscale Res Lett. 2014 Aug 15;9(1):397. doi: 10.1186/1556-276X-9-397 (PMC4144986; doi:10.1186/1556-276X-9-397)
Supplement: Additional file 1 — Supplementary figures. A document showing six supplementary figures showing UV–Vis absorption of RNase A, PL and XPS spectra of C-dots, and influence of ratio reactants, reaction time, carbon sources, and surface modification molecules on the PL character of RNase A@C-dots. [file 1556-276X-9-397-S1.docx]

**Additional file 1**

**

**

**Figure S1** UV-vis absorption of RNase A (black line) and RNase A under microwave for 4 min (red line).


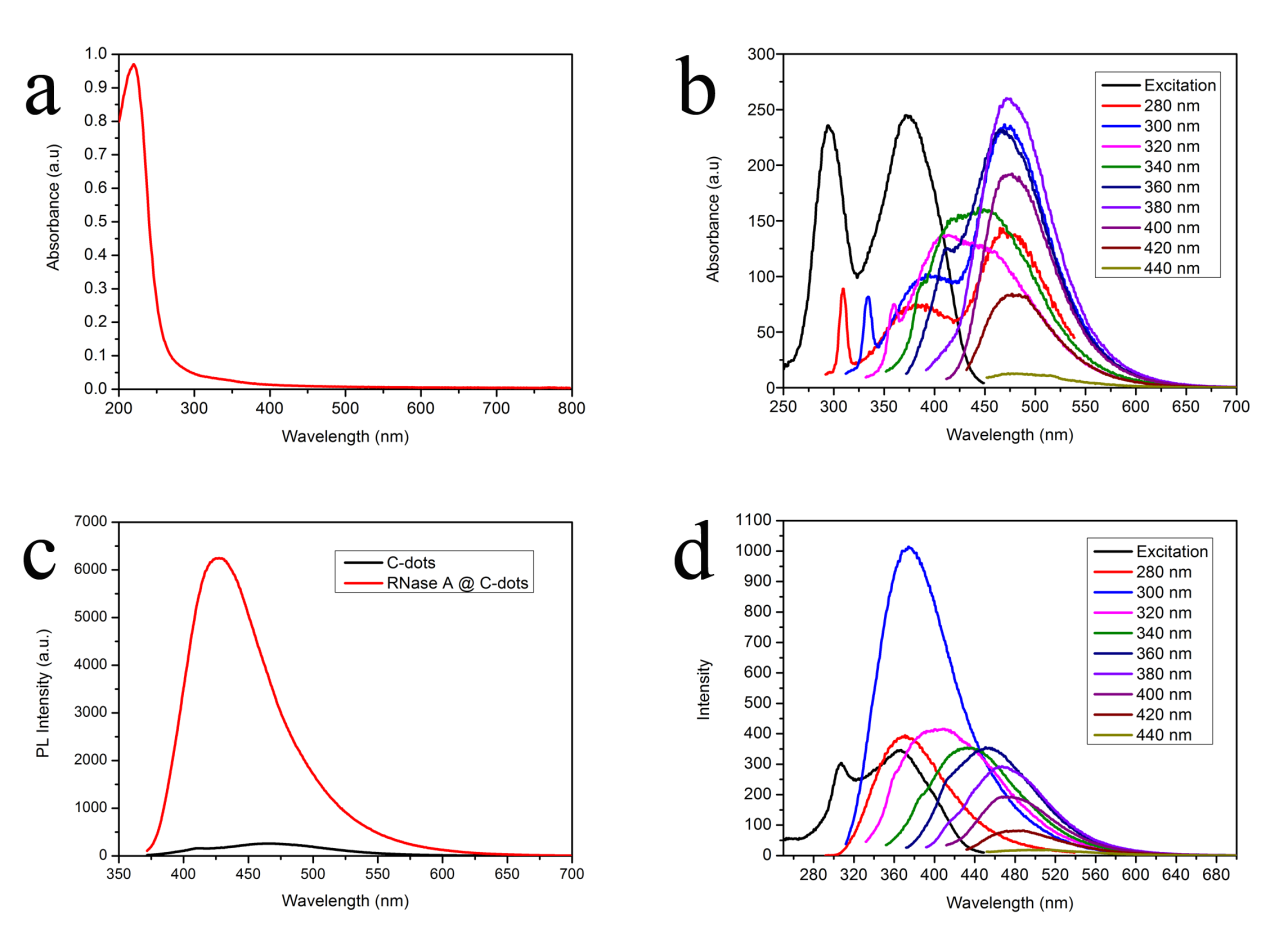


**Figure S2** a) UV-vis absorbance of C-dots. b) PL spectra of C-dots at excitation from 280 to 440 nm in 20 nm increment. c) PL intensity excited at 360 nm of C-dots (black line) and RNase A @ C-dots (red line). d) PL spectra of C-dots mixed with RNase A for 24h at 60℃ at excitation from 280 to 440 nm in 20 nm increment.


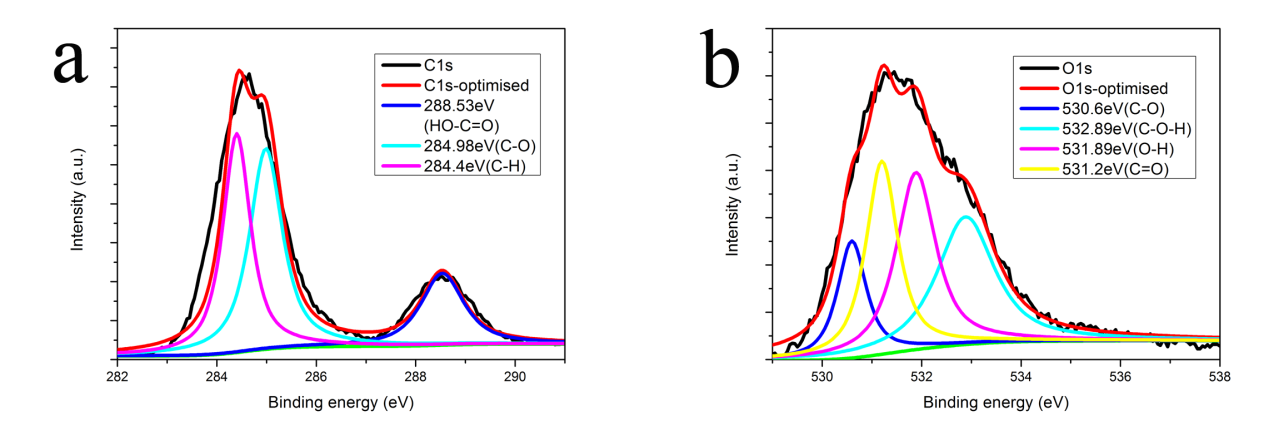


**Figure S3** a) XPS C 1s spectrum, b) XPS O 1s spectrum of C-dots.


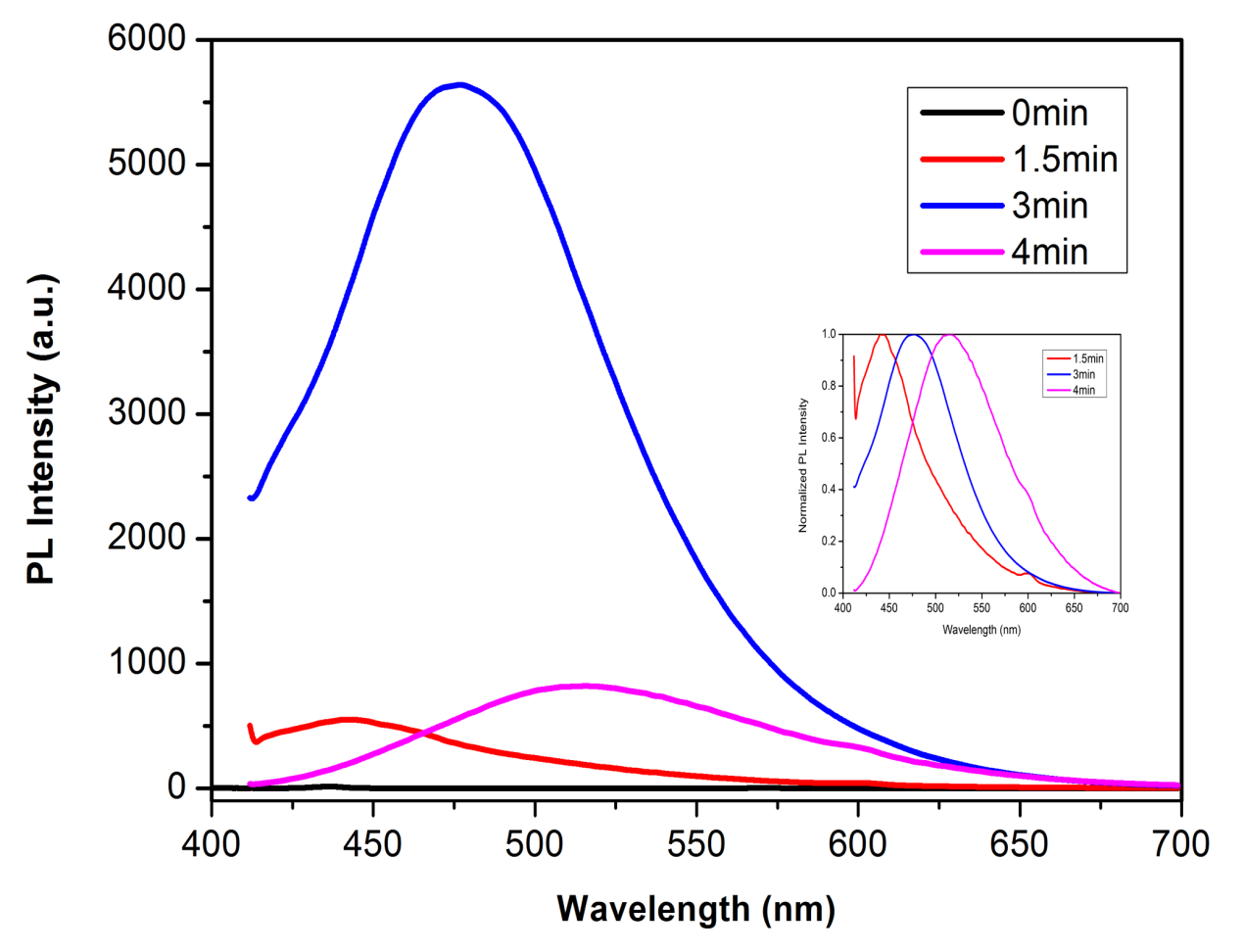


**Figure S4** PL spectra of C-dots under microwave for 0, 1.5, 3, and 4 min, excited at wavelength of 380nm .


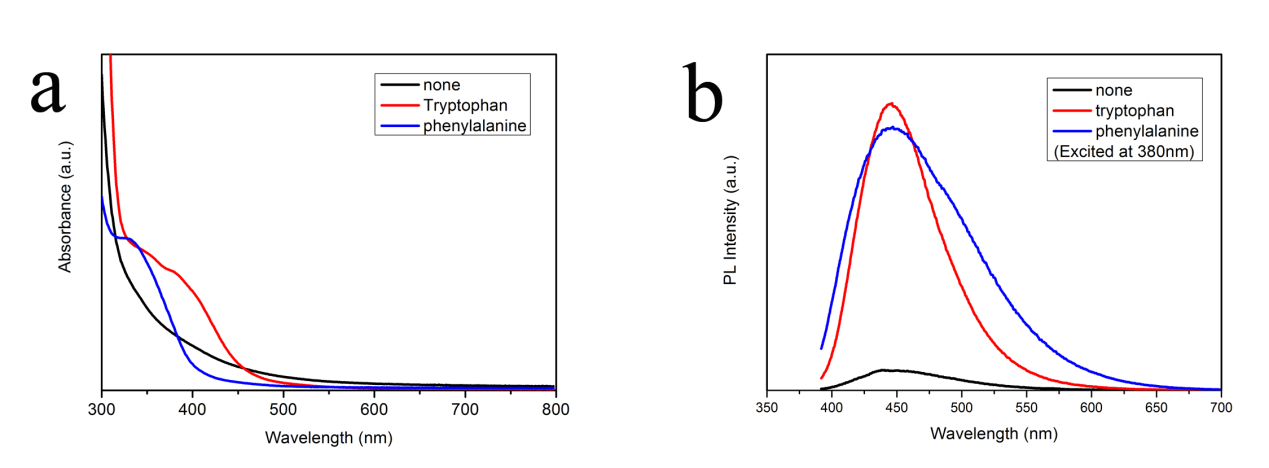


**Figure S5** a) PL spectrum of C-dots (none), C-dots pssivated by tryptophan and C-dots passivated by Phenylalanine. b) PL spectra of C-dots1(none), C-dots pssivated by Tryptophan and C-dots passivated by Phenylalanine, excited at wavelength of 380nm.


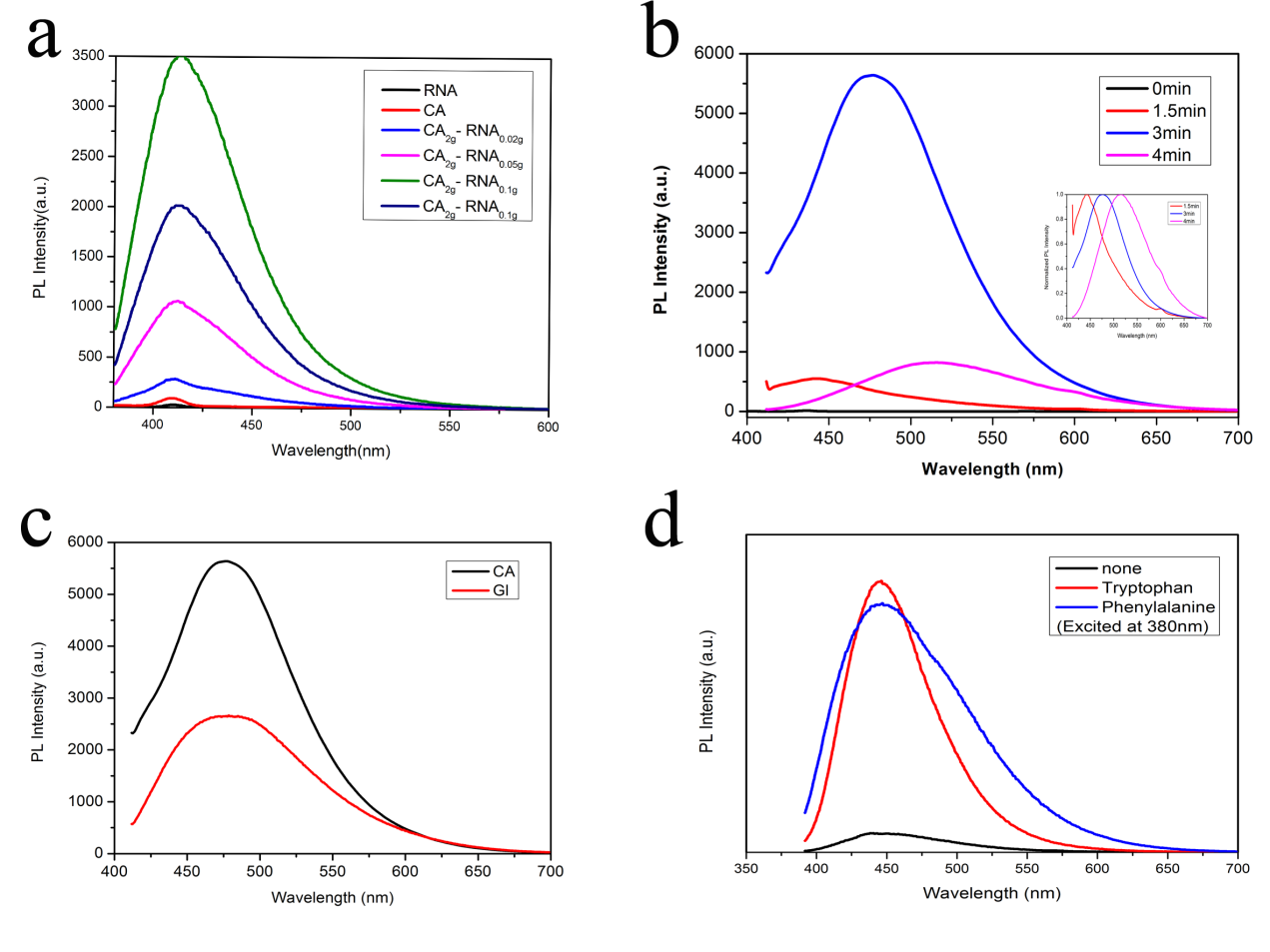


**Figure S6** The influence of a) Ratio of reactants b) Reaction time c) Carbon resources d) Surface modification molecules on the PL character of RNase A@C-dots
